# MultiSig: a new high-precision approach to the analysis of complex biomolecular systems

**DOI:** 10.1007/s00249-013-0924-y

**Published:** 2013-08-29

**Authors:** Richard B. Gillis, Gary G. Adams, Thomas Heinze, Melanie Nikolajski, Stephen E. Harding, Arthur J. Rowe

**Affiliations:** 1 National Centre for Macromolecular Hydrodynamics, School of Biosciences, University of Nottingham, College Road, Sutton Bonington, LE12 5RD UK; 2 Center of Excellence for Polysaccharide Research, Institute of Organic Chemistry and Macromolecular Chemistry, Friedrich Schiller University of Jena, Humboldtstraße 10, 07743 Jena, Germany

**Keywords:** Sedimentation equilibrium, Fitting algorithm, Rayleigh interference optics, Molecular weight histograms, Point average molecular weights, Spatial filtering

## Abstract

MultiSig is a newly developed mode of analysis of sedimentation equilibrium (SE) experiments in the analytical ultracentrifuge, having the capability of taking advantage of the remarkable precision (~0.1 % of signal) of the principal optical (fringe) system employed, thus supplanting existing methods of analysis through reducing the ‘noise’ level of certain important parameter estimates by up to orders of magnitude. Long-known limitations of the SE method, arising from lack of knowledge of the true fringe number in fringe optics and from the use of unstable numerical algorithms such as numerical differentiation, have been transcended. An approach to data analysis, akin to ‘spatial filtering’, has been developed, and shown by both simulation and practical application to be a powerful aid to the precision with which near-monodisperse systems can be analysed, potentially yielding information on protein-solvent interaction. For oligo- and poly-disperse systems the information returned includes precise average mass distributions over both cell radial and concentration ranges and mass-frequency histograms at fixed radial positions. The application of MultiSig analysis to various complex heterogenous systems and potentially multiply-interacting carbohydrate oligomers is described.

## Introduction

The analytical ultracentrifuge (AUC) is an instrument that subjects solutions of macromolecules to high centrifugal fields (up to 300,000×*g*) and explores, via a range of optical modes of analysis, the resultant re-distribution of solute particles. Its use complements and is orthogonal to other techniques, in particular to light-scattering (dynamic or static) and/or column-based methods (SEC, SEC-MALS). Major advantages of AUC methods are that matrix-interaction effects (as with SEC or other columns) are not present, and the optical signal recorded for a given solute mass concentration is invariant with respect to solute particle size, unlike light-scattering systems. The dynamic range of solute size susceptible to AUC analysis is vast: from a few hundred daltons to tens of mega-daltons.

There are two modes of AUC analysis: sedimentation velocity (SV) and sedimentation equilibrium (SE). In SV mode the solute particles are progressively pelleted over a period of time, during which the solute undergoes diffusion in addition to migration under the centrifugal field. Multiple (often 100+) optical scans of the AUC cell are recorded, from which solute distributions can be derived. The two optical systems most widely used employ (1) absorption optics at wavelengths (200–600 nm) user specifiable or (2) interference fringe optics, based upon the refraction index increment with respect to solvent of the solute. For the former, the solutes under analysis there must possess a usable chromophore; for the latter it is convenient that all solutes have refraction increments that differ only slightly with respect to solute type (e.g. protein or carbohydrate) and even less within those types.

Sedimentation equilibrium is a technique based upon sound thermodynamics and as such has been seen as a ‘gold standard’ for the characterisation of the size and interactions of macromolecular solutes. It is simple to perform: solutions held in a constant centrifugal field attain after a period of time distributions that are time-invariant when the sedimentation potential exactly equals the chemical (diffusive) potential at every position in the AUC cell. Analysis of such distributions can then yield estimates for important solute-related parameters, such as range of solute sizes present, their weight values and possible interactions. The basic data set recorded by the highest precision optical system is a set of fringe “increments” (*j*_*r*_) over 200+ radial values (*r*). Precision in individual *j*_*r*_ values is ~0.005 fringe in normal practice, which with the total signal being ~1+ fringe suggests that the information content of the data set must be very high. In specific areas where full advantage can be taken of this high precision in recorded data then it is easily possible to define complex protein interactions at a level that equals or exceeds that given by any other biophysical technology (Rowe 2011).

In general, however, the method has suffered from three serious limitations: (1) for most computational purposes, the relative fringe increments (*j*_*r*_) need to be replaced by absolute fringe increments (*J*_*r*_), where *J*_*r*_ = *j*_*r*_ + *E*; where *E* is an unknown ‘baseline offset’ (Harding 2005); (2) the methods used for computing average molecular weight values routinely involve numerical differentiation of the data set, an inherently ‘noisy’ procedure. We have sought to re-examine these issues and have defined a novel approach (MultiSig), which on the one hand yields high precision estimates for solute-solvent interactions of monodisperse solutes and on the other hand yields solute size distributions together with the definition of any algebraically definable average mass value at any point in a radial distribution at levels of precision not previously attainable.

## The MultiSig algorithm

Our approach starts with the observation that for any solute distribution at equilibrium and, in the absence of specific interactions (i.e. at low concentration), the final distribution of concentration or fringe values (*J*_*r*_) with respect to radius (*r*) is given by

1 $$ J_{r} = \sum\limits_{i = 1}^{i = n} {c_{i} J_{\text{ref}} } \exp [0.5\sigma_{i} (r^{2} - r_{\text{ref}}^{2} )] $$

for a system of a large number (*n*) of components, where σ_*i*_ is the reduced flotational mass of the *i*th component, and *J*_ref_ is an initial, estimated reference value at a radial position *r*_ref_. This estimate serves simply as a scaling constant in the *c*_*i*_ values, and the product *c*_*i*_ *J*_ref_ is a measure of the amplitude of the weight seen at the particular σ_*i*_. This reference position can be at any position in the cell: often a useful radial value to employ is that of the experimentally observed or ‘consensus’ hinge point, easily found from radial scans during the approach to equilibrium.

The averages evaluated found at this radial value can, for low speed equilibrium, be related to the solution as loaded into the cell. The parameter σ is defined by

2 $$ \sigma = M(1 - \overline{v}_{i} \rho_{\text{solv}} )\omega^{2} /RT $$

where *M* is the molecular weight of the solute, *R* the gas constant, *T* the temperature (K), $\overline{v}_{i}$ the partial specific volume of the solute component, ρ_solv_ the density of the solvent and ω the angular velocity (rad/s) of the rotor. For other than simulated data sets a baseline offset (*E*) as defined earlier must be added. This reduced flotational weight (σ) is, rather than the molecular weight (*M*), the actual parameter that is yielded by the SE method: and all averages of all types are referred to it.

We could, theoretically, using classical non-linear least-squares algorithms, fit an empirical (or simulated) data set [*J*_*r*_, *r*] to a function of the type given by Eq. (1) employing *n* terms at equal intervals in the argument (σ_*i*_), where *n* is a number equal to or larger than the number of solute components. This procedure of non-linear fitting may be regarded as the use of an operator (the NLF operator) under which a mapping from the data set [*J*_*r*_, *r*] to a data set [*c*_*i*_(σ_*I*_), σ_*I*_] is effected. The latter set can then be used to define (1) the weight distribution of the system [a simple plot of *c*_*i*_(σ_*I*_) vs. σ_*i*_] and (2) the principal averages (number, weight and *z*) from standard formulae:

$$ \sigma_{n} = 1/\left\{ {\sum\limits_{i = 1}^{i = n} {(c_{i} /\sigma_{i} )} /\sum {c_{i} } } \right\}\quad \sigma_{w} = \sum\limits_{i = 1}^{i = n} {(c_{i} \sigma_{i} )} /\sum {c_{i} } \quad \sigma_{z} = \sum\limits_{i = 1}^{i = n} {(c_{i} \sigma_{i}^{2} )} /\sum {(c_{i} \sigma_{i} )}.$$

Thus, in principle at least, these three averages—or indeed any other which can be defined by the set [*c*_*i*_(σ_*i*_),σ_*i*_]—can be computed for a given radial value (*r*) or a given radial concentration (*J*_*r*_) without resorting to numerical differentiation procedures, which have to date been regarded as unavoidable. Furthermore, the distribution *c*_*i*_ = *f*(σ_*i*_) should describe the distribution of weight values within the total solute.

However, it is clear that the problem with applying the above algorithm lies in the fact that *n* will be an unknown and (for polydisperse systems) a potentially very large number. To circumvent this problem, we have taken advantage of the well-known fact that the fitting of multiple exponential terms, where the exponents are closely related, is a notoriously ‘ill-conditioned’ procedure. This does not of course mean that data defined by the summation of multiple exponential terms cannot be fitted using standard procedures: it means that the values of the individual exponents generally cannot be retrieved with any degree of precision (see the review by Petersson and Holmström 1998). In other terms, the mapping from the data set to the parameter set under the NLF operator is one-many. Our solution to this problem has been to actually take advantage of this ‘ill conditioning’. We fit data sets to a function (MultiSig) defined by the summation of a series of 17 exponential terms, where the value of each *i*th exponent is defined by

3 $$ \sigma_{i} = 1.15\sigma_{i - 1} . $$

This spacing of the values of successive exponents, which is logarithmic as defined by Eq. (3), is chosen—and has been confirmed by detailed simulation—such that any attempted resolution of their values would fail (i.e. would be wholly ill conditioned). The choice of 17 terms is dictated by practical computational considerations: fitting data to a multi-exponential series is a slow process, and the employment of a larger set of terms would render the programme—which routinely takes 20–30 min to run—impossibly slow.

The MultiSig equation, as fitted, is then given by

4 $$ j_{r} = \sum\limits_{i = 1}^{i = 17} {c_{i} j_{\text{ref}} } \exp [0.5*0.5\sigma_{i} 1.15^{(i - 1)} (r^{2} - r_{\text{ref}}^{2} )] + E $$

where an initial value of σ_*I*_ is user-specified. The range in σ extends from 0.5σ_*I*_—from whence the second term 0.5 derives—to 4.48σ_*I*_, and this dictates the range of solute sizes that can currently be accommodated. A ‘starting set’ of parameter values must be supplied—and we routinely use a ‘typical log-normal distribution’ set—for all systems, including analysis of single-component, monodisperse proteins. This we have validated by numerous simulations. MultiSig is a Pascal language scripting plug-in written within the general curve-fitting and plotting programme ProFit™ (Quantum Soft, Zürich), implemented under Apple OS-X using an **Intel** ^®^ Core™ **i7** Processor. The output file is a spreadsheet (whose contents can be pasted into MS-Excel if required) summarising the input data and all computed parameters, including the estimates for number, weight and *z* averages, for *E* and for the individual estimated *c*(σ_*i*_) sets and the computed average set derived from the latter.

Because of the one-many nature of the mapping from data set to parameter set, there can ‘never be the same fit twice’. We thus routinely take a series of fits—usually 10–20—and average the output final parameter sets ([σ_1_ − σ_17_], σ_*n*_, σ_*w*_, σ_*z*_, *E*). The fitting procedure, which we have previously used in other contexts (Ang and Rowe 2010), involves an initial ROBUST fit followed by a Levenberg-Marquardt fit. The latter is not a suitable ‘first fit’ routine, as with poor initial estimates of parameters, singularities are frequently encountered. Although the improvement of fit resulting from the second fit is marginal, there is little time penalty involved, so this is our routine procedure. For each initial fit the starting set of parameters is randomised by use of a routine that applies normal random percentage variation to each parameter individually. As all the σ_*I*_ values floated are constrained to be positive algebraically, the use of percentage variation (usually ±7 % but can be user-specified) minimises the danger of the programme being terminated because of algebraic invalidity of the values supplied by the randomisation routine.

The final output of MultiSig is a table of individual and averaged values for the three principal averages, for the baseline offset (*E*) and for the 17 coefficients. A plot of the latter (averaged) amplitudes against σ gives a profile that is an estimation of the size (weight) distribution of the sample at the radial position selected. This plot of *c*(σ) vs. σ can readily be transformed into a plot of *c*(*M*) vs. *M* via Eq. (2), provided that all components of the sample have the same partial specific volume.

We currently restrict application of the MultiSig algorithm to data acquired at low speed equilibrium (σ < ~10). This is partly because to date we only have, through simulation, validated MultiSig under these conditions—and partly because if a study of a polydisperse system is intended to return meaningful average weight values referred to the system as loaded, then high speed analysis will be liable to distort the distribution by selective depletion or even removal of higher mass species.

An extension of the MultiSig programme, MultiSig_radius, allows for a user-specified series of fits to be performed at fixed radial intervals. As this programme would take an unfeasible length of time to run if every fit was to be over 20 fit averages, the programme MultiSig_radius employs only a single fit at each radial position. Although this is non-optimal, the noise level is so very markedly superior to earlier algorithms employing numerical differentiation procedures that it is acceptable. It should be kept in mind that finding output values for σ_*n*,*r*_, σ_*w*,*r*_, σ_*z*,*r*_ over a series of radial values, which can be fitted to a trend line, is itself a noise reduction (smoothing) procedure.

Based upon simulation, the potential importance of knowledge of radial point-average values of σ for characterising complex macromolecular systems has long been appreciated (Roark and Yphantis 1969; Teller 1973). However, it has been very clear that in practice, using the analytical techniques then available (twofold pre-smoothing of raw data prior to numerical differentiation), the resultant noise level in output values was too high for quality interpretation. Moreover, probably due to over-smoothing, apparent trends appeared in the output value set that had no genuine basis in reality (Teller 1973).

## Methods

### Sedimentation equilibrium experiments

These were carried out in a Beckman-Coulter AUC using the ProteomeLab control system at speeds and for durations selected with the aid of locally written software (SE_Speeds.xls). Radial scans were logged using Rayleigh fringe optics. Four initial and four final scans at equilibrium were logged and averaged. The final data set selected for analysis was obtained by subtraction of these two averaged data sets, removing any points near the meniscus or the cell base where evidence of re-distribution could be detected. This procedure (Ang and Rowe 2010) largely eliminates baseline gradients or other irregularities in the trace, and is superior to methods based upon use of a dummy cell. The baseline offset (*E*), defined as uniform in value over the selected radial interval, cannot however be eliminated in this way.

Corrected data sets have been analysed using the MultiSig programme, with a starting value for σ_*I*_ that should be in the region of, for choice slightly below, the expected mid-region of σ values. An initial fit using only two iterations is performed and the distribution of σ values inspected. If need be, the σ_*I*_ employed in the final 20-iteration fit is amended. The criterion for a ‘good’ value is that the final distribution of σ values should be wholly within the window (from 0.5σ_*I*_ to 4.48σ_*I*_) used by the programme.

The precision of the final profile, which normally employs only 17 values for σ on the *x* axis, can be improved to a degree by carrying out the MultiSig fit two extra times, with two extra values for the starting value for σ producing a logarithmically interpolated set of 3 × 17 = 51 *x* values in the distribution. Only three iterations are now employed for each fit, to keep the total compute time manageable. An example of this modified MultiSig procedure is shown below (Fig. 3).

The radial-dependence programme MultiSig_radius is normally only employed on a system after it has been characterised using MultiSig. Thus the choice of initial σ_*I*_ value is trivial.

Solutions of chicory root inulin were prepared by direct dissolution of the powdered product (kindly donated by Kelloggs UK) into 90 % aqueous DMSO. Solute concentration was checked using a digital refractometer (Atago DD-5).

### Generation of simulated data sets

We have followed our normal routine in generating data sets via the Tabulate function within ProFit™ (Ang and Rowe 2010). Normally distributed random error is added via a locally written plug-in (AddError): our default option is to use ±0.005 fringes, this being at a level normally found in practice with fringe optics (Ang and Rowe 2010). We have also computed parameter values from simulated data with ±0.002 fringe precision, these being the approximated limit to which error in Raleigh interference fringe data in the AUC might potentially be reduced (Ang and Rowe 2010). As MultiSig returns estimates for point-averaged σ_*I*_ values at defined radial position, it is convenient for testing purposes to be able to compute those averages from the initial (i.e. no added error) data set. This is simple to perform algebraically for polydisperse systems if the simulated data are generated by the addition of the members of a set (of any size) of multiple discrete components. In all cases the simulation was of fringe optical data. Normally 200 data points were generated over a 2-mm column length. Although this is a ~2 times smaller data set than is yielded experimentally by the XL-I, the restriction is necessary to avoid unfeasibly long programme run times. Our findings will thus be conservative with regard to the level of precision to be expected in returned parameters when real data, with many more data points, are analysed.

## Results

### Analysis of polydisperse systems

**Fig. 1:**
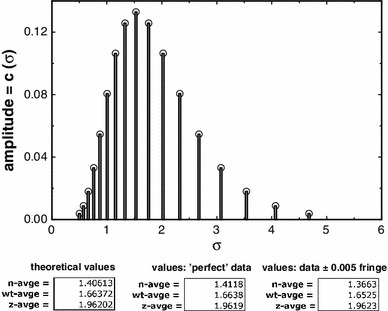
Plot of simulated data values for a log-normal distribution (*vertical lines*) together with values retrieved by the MultiSig programme (*circles*). Theoretical values are given, together with the results yielded for estimates of the principal averages, and with the errors found (which would be subject to slight stochastic variation in results from repeated runs of the software). The effect of addition of normal random error to the simulated data is also shown, as are the two conventionally calculated ratios of averages, or ‘polydispersity indices’ (*bottom*)

We have simulated a data set (no error) for a polydisperse system of log-normal distribution, total load concentration 1 fringe, column length 2 mm (6.9–7.1 cm), at low speed equilibrium. In the absence of simulated error, the retrieval of the input parameters [*c*(σ) vs. σ] and of the theoretically computed averages is close to perfect (Fig. 1). The ‘baseline offset’ (*E*) is retrieved with a precision of <2e−6 fringe. As an additional check on this finding, we have undertaken the same fit using a FORTRAN routine (NAG Fortran Library Routine E04JYF) on a different platform, and this shows complete agreement. When normal random error at ±0.005 fringe is added to the simulated data set we can still demonstrate retrieval of number- (–2.8 % error), weight- (−0.6 % error) and *z*-average (−0.01 % error) values for a fixed radial position (Fig. 1). It is notable is that errors in the *z*-average values are very small indeed. The error in *E* is now appreciably greater (±0.005 to ±0.010) than it is in the absence of simulated error, to an extent depending on the particular data set, or on the number of the iteration. Such an error level is acceptable.

A surprising fact is that the estimates for the *z*-average values, generally regarded as the most difficult of all to define at any level of precision, are by this methodology the most precise of the three estimates.

**Fig. 2:**
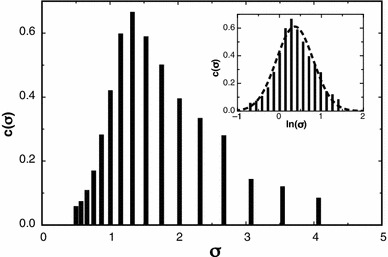
The figure profile obtained by MultiSig analysis of data from an SE run on a sample of pullulan. The *inset* profile shows the data plotted on a logarithmic scale, showing a normal Gaussian distribution

Can data from a real polymer system yield data at the same level of precision as in the above simulation? To test this, we have analysed data from an SE run on a sample of the well-defined standard polysaccharide pullulan of stated molecular weight 404 kDa and polydispersity ratio *M*_*w*_/*M*_*n*_ = 1.13 (Polymer Laboratories, sample batch no. 20907-2). The results show clearly that a log-normal distribution is given in the *c*(σ) vs. σ profile (Fig. 2) and the estimated molecular weight at a radial value estimated as being close to the consensus hinge point and using a value for the partial specific volume = 0.602 ml/g (Kawahara et al. 1984) is 390 kDa, in good agreement with stated value, as was the polydispersity ratio, estimated as 1.13.

**Fig. 3:**
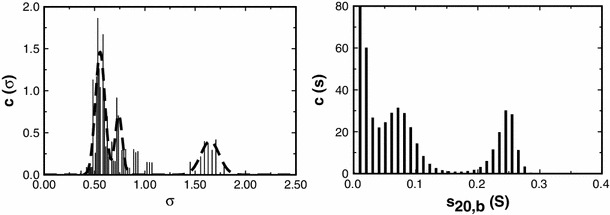
Plot of *c*(σ) vs. σ (*left*) and of *c*(*s*) vs. *s*_20,b_ (*right*) distributions computed via the routines MultiSig and SEDFIT respectively from the equilibrium distribution of a sample of the polysaccharide chicory root inulin at cell loading concentration of 1.0 mg/ml. For the MultiSig analysis three fits were performed, with two additional starting σ values interpolated logarithmically between the 17 starting σ values normally employed (see “Methods”)

We have undertaken MultiSig analysis on a range of other polydisperse biopolymers. Values yielded for the principal averages are routinely of the magnitude expected, based upon different techniques. A particular feature of the MultiSig output however is that the profile of *c*(σ) vs. σ yielded can be compared with a plot of *c*(*s*) vs. *s* or ls_g*(*s*) vs. *s* as computed by SEDFIT (Schuck 2000) from a sedimentation velocity study of the same sample (*s* is the sedimentation coefficient). We illustrate this for a sample of inulin (Fig. 3).

The overall shape of the two distributions is similar: both show a component having a narrow distribution (σ = 1.65, *s*_20,b_ = 0.255S) and a series of lower *σ*/*s* value minor components, which may well be degradation products (Windfield et al. 2003). There is no valid way in which the bi- (or possibly tri-) modal *c*(*s*) distribution (right) can be transformed to yield even the relative masses of the two components, whereas with an estimate (0.60 ml/g) for the partial specific volume of the solute available, the (average) absolute molecular weights of the two species can be defined from the σ values associated with the peaks and their relative concentrations obtained even without that knowledge. Inulins, widely studied and used, usually have a major component with a molecular weight in the region of 5–6 kDa, sedimentation coefficient *s*_20,w_ ~0.7S (Imran et al. 2012; Azis et al. 1999). They readily degrade to smaller species, particularly at acidic pH levels (Windfield et al. 2003). The value for the molecular weight of the (higher molecular weight, putatively intact) component is 5.9 kDa—within the accepted range quoted—and for the two lower weight species identified (Fig. 3, left) estimates of 2.65 and 1.99 kDa are computed. A comparison with the results from the *c*(*s*) profile (Fig. 3, right) is difficult, inasmuch as we are working at very low *s* values and are dependent on near-meniscus data and on the precise estimated position of the meniscus. There is however good qualitative agreement between the results from the two methods, if we are prepared to reject the exact (ultra-low, i.e. <0.05S) values for the lowest *s* value ‘components’. Perhaps surprisingly, however, the results from the *c*(σ) vs. σ (Fig. 3, left) do show that those ultra-low *s* value ‘components’ seen in the *c*(*s*) profile are real, albeit with apparent *s* values that are not reliable.

### Simulation of the use of the MutliSig_radius routine

**Fig. 4:**
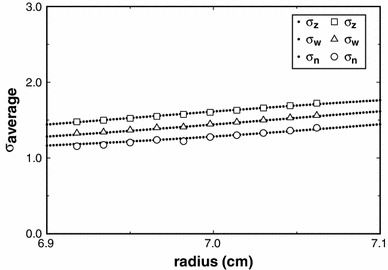
Plots of the estimates yielded for three principal averages using the routine MultiSig_radius applied to simulated data for a 1–1 monomer–dimer mixture, where σ_monomer_ = 1. The ‘*continuous*’ (*dotted*) *lines* are specified by the absolute algebraically computed values expected, whereas the *open symbols* are the results from the MultiSig_radius routine, applied to data of ±0.005 fringe precision, total cell load 1 fringe

We now consider the use of the MultiSig_radius routine for the analysis of polydisperse systems to explore the level of precision that would be expected from the analysis via a MultiSig_radius of a simple two-component 1–1 mixture of monomer and dimer, employing only a single estimate for the average *s* value at each radial position. Simulated data sets have been computed, based on (1) machine precision, (2) ±0.002 fringe or (3) ±0.005 fringe. Figure 4 shows the results from a MultiSig_radius *n* analysis of a simulated data set.

It is clear that for the last of these sets, i.e. the ‘worst case scenario’, the σ_*z*_ and σ_*w*_ values are very close to theoretical, and this also was so for cases (1), (2) and (3), not shown. However the σ_*n*_ values with a precision of ±0.005 fringe show a limited degree of both scatter and systematic deviation, as would be expected from the results yielded for a simulated log-normal distribution (Fig. 1). For practical work, the agreement between theoretical expectation and actual output is at a high level, certainly adequate for most practical purposes.

### Analysis of oligodisperse systems

This is an interesting area, which we are currently studying in some detail. There is the potential using the MultiSig_radius routine for defining not only the range of species present in a complex system, but also for gaining insight into any reversible equilibria that may be present. In a study of a range of aminopolysaccharides (Heinze et al. 2011), we have demonstrated that a pseudo-monomeric state can exist, apparently close to, but probably not truly, monodisperse. This ‘monomer’ was shown to be capable of assembling to a series of oligomeric states. We have continued our analysis in this area and now report results from another aminopolysaccharide, M902TODA.

**Fig. 5:**
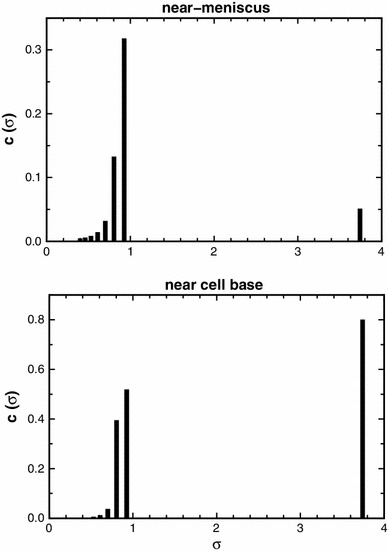
*c*(σ) vs. σ profile computed by MulitSig analysis for the aminopolysaccharide M902TODA for cell radial positions near to the meniscus (*upper plot*) and near to the cell base (*lower plot*)

Our general approach has been to take the SE experiment up to a rotor speed where components higher than small oligomers will be mostly or entirely pelleted. This enables us to ‘probe’ the state of association of lower molecular weight species. We achieve this by specifying in MultiSig cell radial positions within the solution column, covering regions close to the meniscus and nearer to the cell base. In Fig. 5 we show that two principal components are present in the *c*(σ) vs. σ profile, with the smaller species predominating in the solution column close to the meniscus, the larger species—approximately 4× greater in weight—increasing markedly as the cell base is approached.

In interpreting these results, it is important to be aware that if any rapid chemical equilibria are present between species, this fact will not be evidenced by the *c*(σ) vs. σ profile, which is simply a histogram showing ‘what species are present’. If however species intermediate in mass are present, then only if they are sufficiently long-lived to contribute to the range of species present on a time-averaged basis would they appear in a *c*(σ) vs. σ profile. The estimated molecular weights for the two species from this profile are 3.85 kDa (from σ = 0.89) and 16.22 kDa (from σ = 3.75), suggesting a monomer-tetramer relationship

These results can be interpreted either in terms of

1. the existence of a dynamic equilibrium between a ‘monomeric’ species and an oligomeric form (probably tetrameric) or
2. a simple mixture of the two forms.

This issue can be resolved by plotting point-average estimates for averages, yielded by the MultiSig_radius, against the fringe number (i.e. concentration) and repeating the procedure for different cell loading concentrations (0.4 and 2.0 mg/ml in the present case). In the case of a reversible equilibrium, present on a time scale short in relation to the duration (1–2 days) of the SE experiment, then all values of the estimates at the two concentrations must lie on a common regression line w.r.t. *J*(*r*). This follows from simple logic: there cannot at any locus for given *K*_a_ value and total solute concentration be two differing equilibrium states (see Teller 1973).

**Fig. 6:**
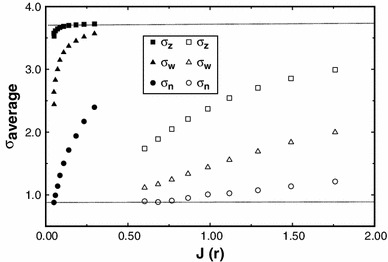
Plot of the three principal averages for a sample of the aminopolysaccharide M902TODA as a function of fringe increment value for cell loading concentrations of 0.4 and 2.0 mg/ml. The *lower* and *upper dashed lines* indicate the values estimated from the *c*(σ) vs. σ profiles (Fig. 5) for the ‘monomer’ and (putative) tetramer, respectively

When we plot the three principal averages for the two different loading concentrations it is very clear that (for this sample at least) there cannot be a reversible, dynamic equilibrium state present (Fig. 6).

These two sets of values do not even come close to being on common regression lines, but rather are typical of a non-interacting mixture. In the limit of two species being present, then at infinitely low concentration only the lower mass species will be found, whilst at infinitely high concentration (infinitely long column) the oligomer species will be totally dominant. There is no simple way in which these extrapolated values can be estimated with any precision, but from the plots shown (Fig. 6) we can say that such values would at least be very consistent with those obtained from the *c*(σ) vs. σ profile (Fig. 5).

### Analysis of mono- (or near-mono-) disperse systems

**Fig. 7:**
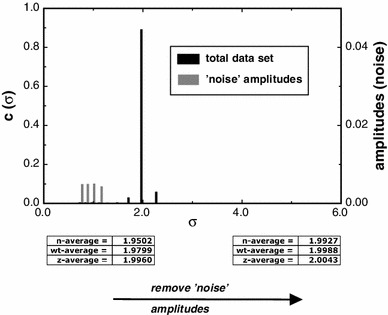
Simulation of the effects of addition of normally distributed random error to a SE data set computed over 200 radial intervals between 6.9 and 7.1 cm for a single component of reduced flotational weight σ = 2.0 on the distribution of *c*(σ) values computed via the MultiSig programme

For systems that are monodisperse (i.e. single component) a MultiSig analysis with ‘17-sigma’ fitting (Eq. [4]) must in theory yield a distribution of *c*(σ) values that is populated in two bins only as a single bin population could only arise if the value of *s* for the solute corresponded precisely with 1 of the 17 values being employed; furthermore the ‘experimental’ parameter values would have to be of infinite precision. Thus in practice, and even for data sets simulated without added error at machine precision, there will almost always be three bins populated, albeit maybe at very low intensity, for one of the bins flanking the main signal. This can be clearly seen in the simulated data (Fig. 7).

However, when significant ‘noise’ is present in the data set, then additional bins in the distributions become populated at low intensity relative to the main peak(s), as evidenced in Fig. 7. This is not surprising. The mapping of the [*c*, *r*] data set onto the *c*(σ) vs. *s* distribution under the NLF operator will be noise sensitive and will result in the appearance of artefactual signal in the lower regions of σ.

Thus we have a situation very similar to that familiar in spectral analysis of noisy images, where a transform (usually a discrete Fourier transform) of the image displays both signal arising from the image in one spectral location and from noise in different part(s) of the spectrum; see Lugt (1964) for the basic theory of ‘spatial filtering’ and Ockleford et al. (2002) for a simple application. Reconstruction of the image—or in our case of the various average σ parameter values—can be achieved by selective employment of only those signal values that are deemed to arise from signal rather than from noise. This general process is termed ‘spatial filtering’. In our case, illustrated in Fig. 7, we employ only the amplitudes (*c*(σ) values) associated with the main peak in the distribution for the computation of the principal σ-averages, ignoring values located in the lower region of the distribution, which have been shown by our simulation to arise from the presence of added noise. The ‘true’ values are well retrieved by means of this ‘spatial filtering’ approach.

**Fig. 8:**
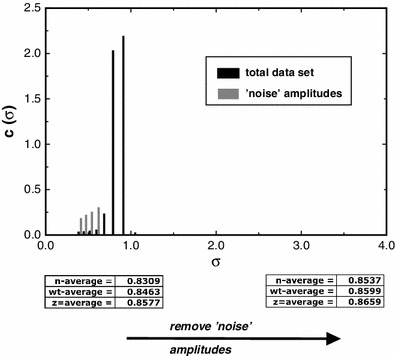
Plot of *c*(σ) (‘amplitudes’) vs. σ for a sample of RNase A, showing the principal averages computed using MultiSig analysis after removal from the computation of peaks identified as ‘noise amplitudes’

This simulated situation we have found to be well mirrored in real data. We have re-analysed data from an RNase A sample whose properties, including both dynamic (*K*_a_) and thermodynamic (2nd and 3rd virial terms), have been fully described (Ang and Rowe 2010). Knowledge of these interaction terms enables us to predict, knowing the partial specific volume, the expected σ_*w*_ value at the concentration (determined from the true fringe value) present at the radial value employed. The results are shown in Fig. 8.

From the data given by Ang and Rowe (2010) the predicted σ_*w*_ value for the monomeric species would be 0.8652, only slightly higher than the value found above (0.8599). We can infer that the value for the partial specific volume of RNAse computed using SEDNTERP and used in the prediction is very close to that yielded by our empirical analysis; however the slight mis-matching is potentially explicable in terms of a difference in the strength of protein-solvent interaction between the assumed and the actual level; see Chalikian (2003) for a general treatment of this topic.

## Discussion

Interest in the analysis of complexity in macromolecular systems is a current area of interest and importance, moving on from and building upon the detailed knowledge that we now have of the solution properties of many purified systems. It has long been realised that SE methods have very real potential for the definition of such complex systems, especially through their ability to define distribution parameters over a concentration range within a single experiment (Roark and Yphantis 1969; Teller 1973). However practical application of these methods has lagged because of the very poor level of precision (often ±20 % or worse) of individual parameters retrieved by the use of numerical differentiation procedures, even after heavy ‘smoothing’. We now show how the fitting of raw data with an infinite series of exponential terms would produce a set of terms from which appropriate summation would yield a set (up to 200+ in number) of point parameter values. This we achieve, in practice and to a very adequate approximation, by taking advantage of the (notorious) ill conditioning of multiple exponential fits for closely related exponential terms; we have constructed a 17-term exponential series (Eq. [4]) in which each term is separated from its successor term by an increment in the coefficient (Eq. 3) of a magnitude that ensures near-total ill conditioning so far as resolution is concerned. The MultiSig programme can average over many fits by random variation of starting estimates for all exponential parameters—essentially a (less usual) form of boot-strapping. However, for study of parameters as a function of fringe value (concentration), a single fit at each locus suffices. By simulation and by application to real data, it is clear that individual values for point-average parameters of a precision never previously approached (e.g. ±<0.1 % for *z*-average) can be retrieved for polydisperse systems, whilst for oligo-disperse systems information on the relationships between identified species can be retrieved.

A significant but almost incidental achievement of the MultiSig algorithm is that for the first time quality estimates (normally <0.01 fringe) can be retrieved for the baseline offset, *E*, and hence that true fringe numbers can now be assigned to all radial positions sampled, without need for any auxiliary practical methodology. This solves one of the oldest problems in the use of Rayleigh fringe optics for SE analysis (Laue 1992; Rowe 2004).

Perhaps surprisingly, MultiSig finds application to the study of systems that are close to monodisperse. Noise removal by a form of spatial filtering is novel and allows for unprecedentedly high levels of precision to be attained in retrieval of σ values (Figs. 7, 8), allowing insight to be attained into the nature of protein-solvent interaction.

There are currently certain limitations on the use of MultiSig. The range of σ values specified should not exceed ninefold (largest to smallest) and should optimally be slightly less. The programme, with 20 iterations, currently takes 20–30 min to run. However, both these limitations are susceptible to solution by the use of a more powerful computing platform, a current topic of investigation. There is thus the expectation that even broader ranges of polydispersity will yield to investigation in the near future, subject to the obvious limitation that for a given SE experiment only a limited ‘window’ of σ (and hence molecular weight values) values can be accommodated for given rotor speed. For mondisperse solutes, sensitivity has now been demonstrated at a level where for a solute (usually protein) of known composition solute-solvent interactions (Chalikian 2003) should be susceptible to delineation in ways not previously possible. All these results have been obtained using simulations and practical data that refer to cell loadings of only 1–3 fringes, hence minimising non-ideality (*c* dependence) effects. Of course, even at solutes present at <0.5 mg/ml such effects cannot be totally absent, and all σ values reported are ‘apparent’; however levels of precision have been attained that make it possible to ascertain *c* dependence effects by direct analysis of multiple samples over a range of concentrations.

Finally, the future implementation of our routines on a much more powerful computer facility will enable the use of a full data set, a wider range of σ values to cover the full ‘window’ of molecular weight values needed and—if needed—the averaging of more iterations in our fitting routine. The full potential of Rayleigh interference fringe optics for the analysis of complex biomolecular systems using SE methodology should then be realised.
